# Lymphangioma Circumscriptum Managed by Intralesional Radiofrequency Ablation: A Case Report

**DOI:** 10.7759/cureus.96349

**Published:** 2025-11-07

**Authors:** Rucha Gore, Anuradha Priyadarshini, Divya Raviprakash, Adikrishnan Swaminathan

**Affiliations:** 1 Dermatology, Sri Ramachandra Institute of Higher Education and Research, Chennai, IND

**Keywords:** frog spawn appearance, intralesional radiofrequency ablation, lymphangioma circumscriptum, minimally invasive, minimal recurrence

## Abstract

Lymphangioma circumscriptum is a benign lymphatic tumor resulting from a malformation in the lymphatic vasculature during the embryonic period, which can present most commonly over the neck or upper trunk. Here, we present a case of a 22-year-old male who came with complaints of multiple grouped papules and fluid-filled vesicles over the right axilla and the right side of the chest since birth. Biopsy and MRI confirmed the clinical diagnosis of lymphangioma circumscriptum. Initially, surgical excision was attempted as a curative option; however, recurrent lesions were observed, prompting consideration of intralesional radiofrequency ablation using the Megasurg Derma India radiofrequency device (Derma India, Chennai, India) as an alternative approach. Four sessions of intralesional radiofrequency ablation were completed. After completion of the sessions, topical 1% sirolimus ointment application was started to prevent recurrence. An excellent response with a 75% reduction in the number of lesions was observed with minimal recurrence. Hence, intralesional radiofrequency ablation may offer a more effective alternative to conventional surgical intervention.

## Introduction

Lymphangiomas are benign tumors resulting from congenital malformation in the lymphatic vessels and account for 4% of all vascular malformations. The main types of lymphangiomas include cavernous lymphangiomas, lymphangioma circumscriptum, and cystic hygroma [1,2]. Lymphangioma circumscriptum presents at infancy but can arise at any age and has no sex predilection. Clinically, lymphangioma circumscriptum presents as multiple grouped vesicles filled with clear lymph, resembling frog spawn, which can appear black, pink, or red due to intravesicular hemorrhage. Common sites of presentation include axillary folds, shoulders, proximal extremities, neck, trunk, oral cavity, and perineum [2]. Usually, the presentation is asymptomatic, but it can be associated with complications like cellulitis, ulceration, and squamous cell carcinoma in long-standing cases [1,2]. Pathogenesis of the disease is explained by Whimster's theory, which postulates that the deep lymphatic cisterns remain connected to the superficial dermal lymphatics and fail to connect with the main lymphatic drainage pathways, due to which the lymphatic fluid accumulates in these malformed channels and subsequently dilates the superficial lymphatics, producing characteristic vesicular lesions [3]. This also explains the recurrence following surgery in which only superficial dermal lymphatics are excised. In this case report, we highlight the use of intralesional radiofrequency ablation as an alternative treatment that targets both superficial and deeper lymphatics, hence reducing recurrence and providing better cosmetic results.

## Case presentation

**Figure 1:**
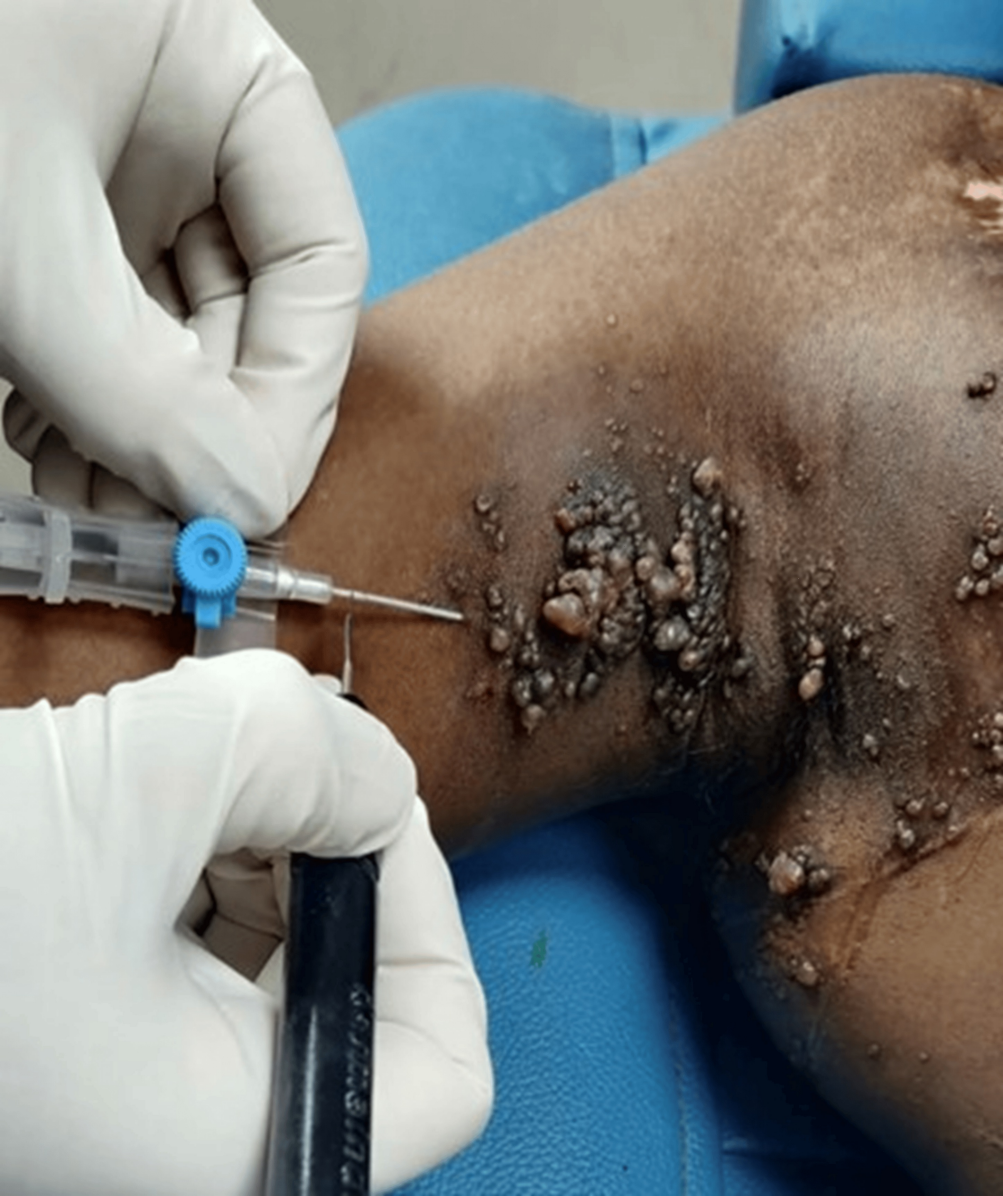
Device used for intralesional radiofrequency ablation.

A 22-year-old male presented with multiple skin-colored grouped papules and vesicles, a few with warty surfaces, resembling the typical frogspawn morphology over the right axilla and right side of the chest since birth, which were sometimes associated with serosanguinous discharge. One lesion over the right axilla was biopsied; on histopathology, the sections showed fibrocollagenous tissue with multiple dilated vascular channels filled with lymph and blood, a few of them having thick walls in the dermis. MRI of the right axilla, right arm, and right side of the chest was done, which showed a thickened subcutaneous plane with fluid signal intensity over the right axilla, right shoulder, and anterior and posterior chest wall. The clinical, radiological, and histopathological picture established a diagnosis of lymphangioma circumscriptum. Differential diagnoses considered included angiokeratoma, viral warts, Kaposi sarcoma, and lymphangiectasia. Angiokeratoma was excluded as the lesions were not dark red to violaceous in color and showed vesicles rather than keratotic papules. Viral warts were ruled out due to a lack of koilocytosis and papillomatosis. Lymphangiectasia, despite showing a similar clinical feature, was ruled out due to the absence of any prior surgery, trauma, or radiation. Kaposi sarcoma was excluded as the patient was immunocompetent and lacked slit like vascular spaces, spindle cell formation, and hemosiderin deposition in the biopsy. Before arriving at our facility, the patient underwent surgical management as a curative option. Despite the surgical treatment, the lesions were found to be recurrent, and the patient expressed apprehension about undergoing further surgeries. Therefore, the team pursued an alternative method of intralesional radiofrequency ablation. In this technique, an 11-blade scalpel was utilized to create a window in the plastic sheath of an 18-G peripheral venous catheter. The intact metallic stylet of the peripheral venous catheter was then contacted with the ball probe of the radiofrequency generator to facilitate energy transmission (Figure 1).

**Figure 2:**
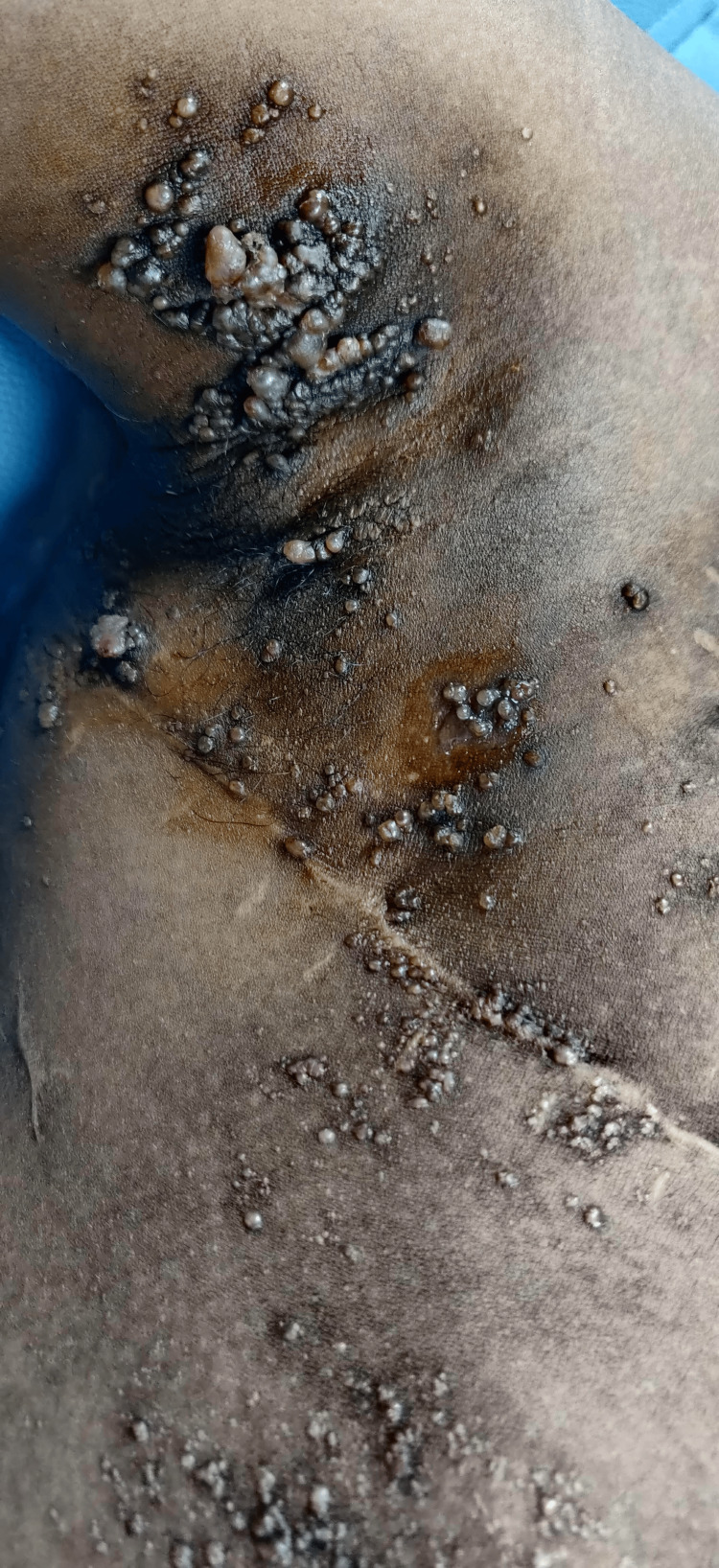
Pre-operative image showing approximately 75-80 vesicles over the right axilla and right side of the chest.

**Figure 3:**
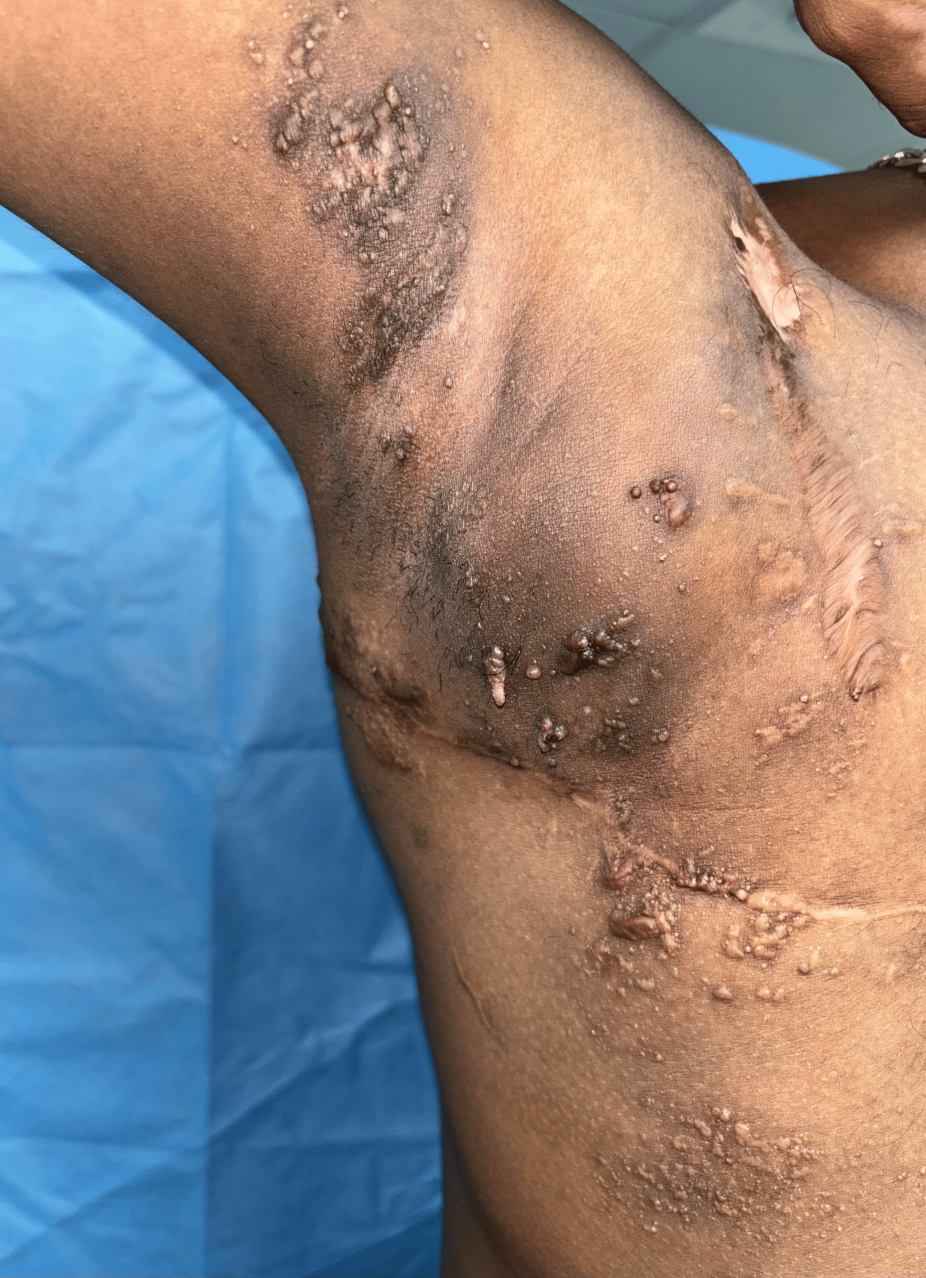
Post-operative image after four sessions of intralesional radiofrequency ablation, showing a reduction in the number of vesicles to approximately 15-20.

The Megasurg Derma India radiofrequency device (Derma India, Chennai, India) was used for the procedure. Procedural consent was obtained from the patient. The procedure was done under local anesthesia using 2% lignocaine. The IV cannula was introduced into the lesion, ensuring that the window remained external to the lesion. The radiofrequency machine was set to a high-frequency coagulation mode, and energy was delivered into the cavity by placing the ball electrode of the radiofrequency probe against the window. The presence of a bubbling sound confirmed that the probe was positioned in the desired plane. Energy was delivered until the bubbling ceased and the surface lesions visibly shrank. After ablating the deeper lesions, the surface lesions were treated using electrofulguration. After each session, the patient was advised to use mupirocin ointment on the skin and was given a prescription for oral amoxicillin-clavulanic acid 625 mg to take for five days to help prevent infections at the treatment site. Four sessions of intralesional radiofrequency ablation were completed, each at an interval of four to six weeks. Following completion of the treatment sessions, the patient was started on topical 1% sirolimus ointment once daily to prevent recurrence of the lesions. The number of vesicles decreased from 75-80 (Figure 2) to 15-20 (Figure 3) in number after four treatment sessions, indicating 70-75% improvement. We followed up with the patient for 18 months. After a year, we treated three to four new vesicles that had recurred over the right axilla with radiofrequency ablation. The patient responded satisfactorily to the treatment, healing with minimal recurrence, cosmetically acceptable scarring, and experiencing slight post-procedure pain and inflammation.

## Discussion

Lymphangioma circumscriptum accounts for 4% of total vascular tumors [1]. Clinically, it can present as microscopic to macroscopic vesicles filled with blood and lymph, which can be associated with multiple complications like pain, ulceration, secondary infection, and lymphorrhoea [4]. During embryonic development, lymphatic cisterns in the deep subcutaneous tissue arising from the lymph sacs fail to connect with the central lymphatic system. Muscle-induced pressure causes sac wall outpouching, leading to the formation of vesicles as observed clinically [2]. As the lymphatic sacs persist deep within the subcutaneous tissue, surgical excision typically removes only the superficial lesions, and the remaining deeper cisterns continue to accumulate lymph due to persistent lymphatic pressure, contributing to lesion recurrence.

Various treatment modalities for lymphangioma circumscriptum have been described in the literature. A combination therapy involving pulsed dye laser and cryotherapy has been employed to treat lesions over the gluteal and femoral region. The hemorrhagic lesions were treated with five sessions of pulsed dye laser at four-week intervals, and yellow translucent lesions were treated with five sessions of cryotherapy at three-week intervals. This regimen resulted in significant regression with few persistent lesions that were managed using additional cryotherapy. Post-treatment, post-inflammatory hyperpigmentation was observed [5]. Sclerotherapy has been described as one of the treatment modalities. In a reported case, intralesional injection of 1% sodium tetradecyl sulfate was administered twice at a three-month interval for lesions on the neck. Approximately 70% improvement was noted, with lesions healing as skin-colored plaques [6]. Topical application 5% imiquimod has been reported as a therapeutic option. In a documented case, lesions over the left shoulder were treated with imiquimod, which was applied on alternate days for a month, resulting in an overall satisfactory response. However, after nine months, few recurrences occurred [7]. Electrodissection has been identified as a treatment option. In another documented case, lesions over the right gluteal region were treated with three sessions of electrodissection at four-week intervals. Post-treatment, the lesions healed with residual, atrophic plaques, and a few persistent vesicles were noted [8]. Surgical excision is considered a definitive treatment option but has a high recurrence of 17-23% [9].

This case was treated using intralesional radiofrequency ablation, a method that delivers an alternating current into the lymphatic cisterns to stimulate collagen formation, thereby obliterating the lymphatic channels and preventing further lymph accumulation, hence reducing the chances of recurrence. First, the deeper lymphatic malformations were ablated to prevent recurrence, followed by treatment of superficial vesicles to ensure cosmesis was maintained. The procedure can be associated with complications like bleeding, infection, burns, and nerve damage, and these can be prevented by using a proper technique, maintaining sterility, and enhancing one’s procedural skills. Despite being a blind procedure, the technique's short learning curve allows one to overcome this challenge. The idea of using intralesional radiofrequency emerged to choose a minimally invasive therapeutic approach, which will provide satisfactory clinical outcomes with minimal adverse effects.

## Conclusions

Intralesional radiofrequency ablation offers a superior treatment option for lymphangioma circumscriptum as compared to conventional surgical techniques, as it is safer, can be performed in an outpatient setting, involves less morbidity, and allows for quicker resumption of the patient’s regular activities. However, this observation is based on a single case; further studies with a larger sample size are required to validate its efficacy and establish it as a standard treatment.
